# Supplementary material for: Liquid-liquid phase separation in supercooled water from ultrafast heating of low-density amorphous ice
Source: Nat Commun. 2023 Jan 27;14:442. doi: 10.1038/s41467-023-36091-1 (PMC9883474; doi:10.1038/s41467-023-36091-1)
Supplement: Supplementary file 1 — Supplementary Information [file 41467_2023_36091_MOESM1_ESM.pdf]

Supplementary Information

Liquid-liquid phase separation in supercooled water from ultrafast heating of low-density amorphous ice

Katrin Amann-Winkel[1,2], Kyung Hwan Kim[3], Nicolas Giovambattista[4,5], Marjorie Ladd-Parada[1], Alexander Späh[1], Fivos Perakis[1], Harshad Pathak[1], Cheolhee Yang[3], Tobias Eklund[1], Thomas J. Lane[6], Seonju You[3], Sangmin Jeong[3], Jae Hyuk Lee[7], Intae Eom[7], Minseok Kim[7], Jaeku Park[7], Sae Hwan Chun[7], Peter H. Poole[8], and Anders Nilsson[1]

[1]*Department of Physics, AlbaNova University Center, Stockholm University, SE-10691 Stockholm, Sweden*
[2]*Max Planck Institute for Polymer Research and Johannes Gutenberg University, 55128 Mainz, Germany.*
[3]*Department of Chemistry, POSTECH, Pohang 37673, Republic of Korea*
[4]*Department of Physics, Brooklyn College of the City University of New York, Brooklyn, NY 11210, USA*
[5]*Ph.D. Programs in Chemistry and Physics, The Graduate Center of the City University of New York, New York, New York 10016, USA*
[6]*SLAC National Accelerator Laboratory, 2575 Sand Hill Road, Menlo Park, California 94025, USA*
[7]*Pohang Accelerator Laboratory, Pohang, Gyeongbuk 37673, Republic of Korea*
[8]*Department of Physics, St. Francis Xavier University, Antigonish, Nova Scotia B2G2W5, Canada*

December 20, 2022

Supplementary Note 1

Thickness distribution of the sample and temperature profile in a slab

The thickness of each individual sample spot is calibrated by comparing the integrated scattering intensity around the first peak of S(q) with the value measured from a sample of known thickness. The thickness distribution is shown in Figure S1(A). The results in the main text were obtained by restricting the analysis to samples of thickness between 40 and 80 μm.

The temperature profile in a slab is simulated and shown in Figure S1(B). The IR laser spectrum at PAL-XFEL is assumed to have a Gaussian profile centered at 2 μm wavelength and a bandwidth spread (2σ or FWHM) of 0.30 μm. The laser profile was convoluted with the absorption spectrum of ice and the average temperature jump is assumed to be 60 K. As shown in Figure S1(B), the temperature profile in the ice slab varies over a range from 50 to 70 K (i.e. 60 K ± 10 K).

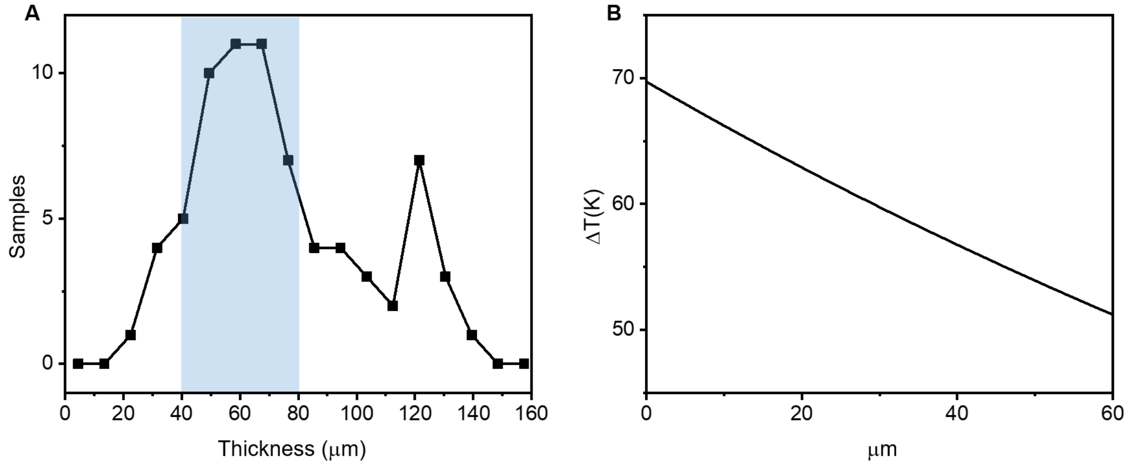

Supplementary Figure 1. (A) Thickness distribution of the sample spots that is used for the measurement. The thickness range used for the main data is indicated in blue. (B) Estimated temperature gradient in an ice slab (thickness=60 μm) as a function of distance.
